# The Role of Videoconferencing Teleconsultation in Improving Transfer Efficiency and Functional Outcomes in Rural Stroke Care: Retrospective Cohort Study

**DOI:** 10.2196/86436

**Published:** 2026-06-04

**Authors:** Chi Sheng Wang, Yi-Ju Chen, Tzu-Chieh Lin, Hui-Mei Huang, Pei-Ru Tu, Po-Lin Chen, Jin-An Huang

**Affiliations:** 1 Division of Neurology, Neurological Institute, Taichung Veterans General Hospital, No. 1650, Taiwan Boulevard, Sec. 4, Taichung City, Taichung, 40705, Taiwan, 886 4-23592525 ext 3325, 886 4-23584403; 2 Telehealth Center, Taichung Veterans General Hospital, Taichung, Taiwan; 3 Institute of Brain Science, College of Medicine, National Yang Ming Chiao Tung University, Taipei, Taiwan; 4 Graduate Institute of Clinical Medicine, National Chung Hsing University, Taichung, Taiwan; 5 Department of Emergency Medicine, Taichung Veterans General Hospital, Taichung, Taiwan; 6 College of Fine Arts and Creative Design, Tunghai University, , Taichung, Taiwan; 7 Department of Post-Baccalaureate Medicine, College of Medicine, National Chung Hsing University, , Taichung, Taiwan

**Keywords:** acute ischemic stroke, rural, teleconsultation, videoconferencing, transfer efficiency

## Abstract

**Background:**

Interhospital transfer delays remain a major barrier to timely reperfusion therapy and are associated with worse functional outcomes in acute ischemic stroke (AIS), particularly in rural regions.

**Objective:**

This study evaluated whether videoconferencing teleconsultation, compared with the standard referral process, was associated with improved transfer efficiency, treatment delivery, and functional outcomes for patients with AIS requiring interhospital transfer in a hub-and-spoke model.

**Methods:**

We conducted a retrospective cohort study of patients with AIS identified as potential candidates for endovascular thrombectomy (EVT) who were transferred from a primary stroke center (PSC) to a comprehensive stroke center (CSC) between January 2022 and December 2024. Patients were managed using either videoconferencing teleconsultation or the standard referral process, defined as telephone-based consultation between emergency physicians at the PSC and CSC, in which clinical evaluation and thrombolysis decisions were made primarily by the PSC emergency physicians. Group allocation was determined via institutional workflow. The primary outcome was door-in-door-out time, with additional analyses on its components. Secondary outcomes included intravenous thrombolysis rate at the PSC, EVT rates at the CSC, door-to-puncture time, reperfusion rates, and 90-day functional outcomes assessed via modified Rankin Scale shift analysis. Safety outcomes included all-cause mortality within 90 days and symptomatic intracranial hemorrhage after intravenous thrombolysis and/or EVT.

**Results:**

A total of 83 patients were included, with 41 (49.4%) in the teleconsultation group and 42 (50.6%) in the standard referral process group (mean age 73.3, SD 12.9 years), and baseline characteristics were comparable. Teleconsultation was associated with a significant reduction in door-in-door-out time (mean 95.2, SD 22.9 vs 132.3, SD 41.5 minutes; *P*<.001) by shortening computed tomography angiography–to-ambulance notification time (mean 44.6, SD 17.4 vs 79.5, SD 37.6 minutes; *P*<.001). The teleconsultation group had higher intravenous thrombolysis rates at the PSC (26/41, 63.4% in the teleconsultation group vs 17/42, 40.5% in the standard referral process group; *P*=.04), higher EVT rates (14/41, 34.1% in the teleconsultation group vs 6/42, 14.3% in the standard referral process group; *P*=.03), and shorter door-to-puncture time (mean 83.0, SD 35.5 vs 118.5, SD 25.9 minutes; *P*=.04) at the CSC. Patients who received teleconsultation demonstrated a greater shift toward better functional outcomes at the 90th day (27/41, 65.9%; odds ratio 4.55, 95% CI 1.96-11.11; *P*<.001) than patients who did not (13/42, 31.0%; odds ratio 1.35, 95% CI 0.63-2.94; *P*=.07). Safety outcomes were comparable between groups.

**Conclusions:**

Videoconferencing teleconsultation was associated with improved transfer efficiency and higher use of reperfusion therapies and was potentially associated with better functional outcomes. This model may represent a feasible strategy for optimizing stroke care pathways in rural settings. Future studies are warranted to assess its applicability in broader stroke populations beyond conventional EVT eligibility criteria across multicenter networks.

## Introduction

Acute ischemic stroke (AIS) treatment is highly time sensitive as delays in reperfusion therapies such as intravenous thrombolysis and endovascular thrombectomy (EVT) significantly reduce their efficacy [1]. The hub-and-spoke model, widely used in AIS management, connects primary stroke centers (PSCs) with comprehensive stroke centers (CSCs) to provide timely access to advanced care [2]. However, this model often encounters delays, particularly at the PSC level. Current guidelines recommend door-in-door-out (DIDO) times within 120 minutes, but many institutions struggle to meet this target [3,4]. These delays significantly impact EVT initiation and worsen clinical outcomes [5].

In rural regions lacking stroke neurologists, telestroke networks have emerged as a promising solution by facilitating remote neurologic expertise [3]. However, despite prior studies demonstrating improved thrombolysis rates with teleconsultation [6,7], its effects on components of DIDO time and long-term functional outcomes remain less understood. Moreover, existing research has largely focused on telephone-based consultation, with limited integration of videoconferencing-enabled real-time assessment, including clinical evaluation, supervision of thrombolysis administration, imaging interpretation, and treatment planning, which may further optimize stroke-related decision-making and enhance interhospital coordination [8,9].

We hypothesized that videoconferencing teleconsultation would shorten DIDO time, optimize stroke-related decision-making, and improve long-term functional outcomes in patients with AIS transferred from a PSC to a CSC.

## Methods

### Study Design and Participants

This retrospective cohort study (December 2024) included patients with AIS identified as potential EVT candidates transferred from a PSC in a rural region of central Taiwan (Puli Branch of Taichung Veterans General Hospital) to a CSC (Taichung Veterans General Hospital). Patients were categorized into teleconsultation and standard referral process groups based on whether real-time videoconferencing for neurologic consultation was used. Demographic characteristics and ancillary tests were assessed upon CSC arrival; the National Institutes of Health Stroke Scale (NIHSS) and modified Rankin Scale (mRS) at presentation to the PSC were scored by emergency physicians in the standard referral process group or by neurologists in the teleconsultation group at the PSC.

The primary outcome was DIDO time, defined as the duration from the patient’s arrival at the PSC to their departure. To identify specific areas of improvement, DIDO time was subdivided into 3 components, as described in a previously validated study [10]: door to computed tomography angiography (CTA; time from arrival to completion of CTA), CTA to notification (time from completion of CTA to notifying the CSC), and notification to departure (time from notification to ambulance departure). Secondary outcomes included intravenous thrombolysis (tissue-type plasminogen activator [tPA], the only thrombolytic agent in our stroke network) rates at the PSC; EVT rates at the CSC; EVT-related factors at the CSC, including door-to-puncture time, reperfusion rate (defined by a modified treatment in cerebral infarction score of >2b), and rate of tPA administration prior to EVT; and 90-day functional outcomes assessed via the mRS. The safety outcomes included all-cause mortality within 90 days and symptomatic intracranial hemorrhage (sICH) after tPA and/or EVT, defined via follow-up brain computed tomography performed 22 to 36 hours after tPA and/or EVT, which was validated by a previous study [11].

### Ethical Considerations

This retrospective cohort study was approved by the Institutional Review Board of Taichung Veterans General Hospital (CE25005B), which granted a waiver of informed consent due to the retrospective nature of the study and the use of existing clinical data as the study posed no more than minimal risk to participants. A deidentified dataset was used for all analyses, and access to the data was restricted to the research team to protect participant privacy and confidentiality. No compensation was provided to study participants. No identifiable participant information or images are included in this manuscript or the supplementary materials.

### Study Inclusion and Exclusion Criteria

The inclusion criteria for this study were as follows: (1) patients clinically diagnosed with cerebral infarction by emergency physicians at the PSC at the time of patient arrival and (2) patients identified as potential candidates for EVT by neurologists in the teleconsultation group or by emergency physicians at the PSC in the standard referral process group.

The exclusion criteria were (1) patients diagnosed with hemorrhagic infarction or other stroke mimics based on brain CTA at the PSC; (2) patients exceeding the EVT time window, defined as 6 hours from onset for anterior circulation symptoms or 24 hours from onset for posterior circulation symptoms, as assessed by emergency physicians in the standard referral process group or neurologists in the teleconsultation group based on international guidelines in place at the time [12] and the reimbursement framework of the Taiwan National Health Insurance system; (3) follow-up period of less than 90 days; (4) patients with missing data; and (5) patients not transferred to the CSC via ambulance.

### The Process of Teleconsultation and Standard Referral Process

**Figure 1.**
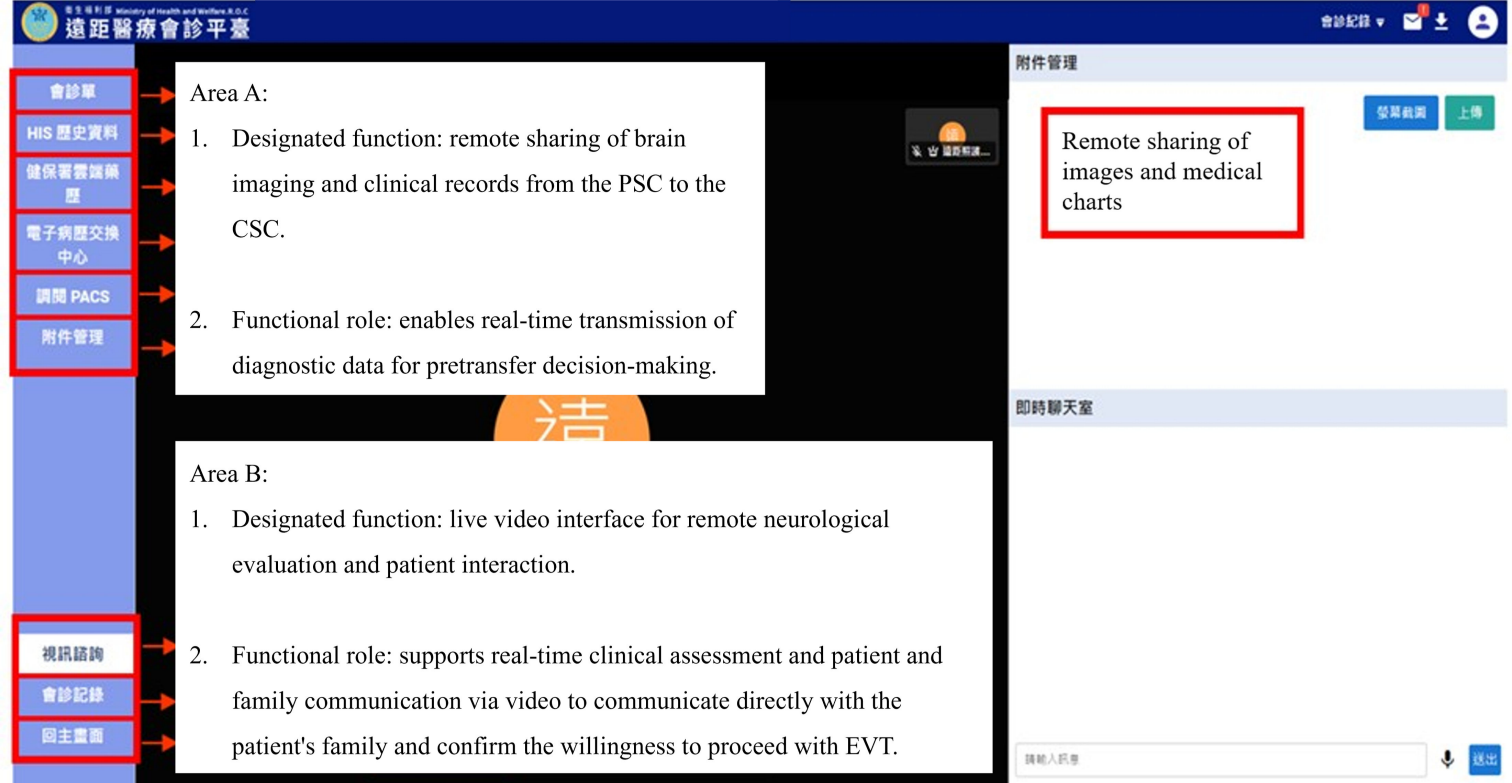
The 5G-enabled teleconsultation interface. CSC: comprehensive stroke center; EVT: endovascular thrombectomy; PSC: primary stroke center.

In the teleconsultation group, the videoconferencing process was initiated upon patient arrival at the PSC. Using a 5G-enabled online consultation system, the neurologist at the CSC accessed shared medical images and charts remotely before or during the brain CTA process. The structural layout and key functional components of the 5G-enabled teleconsultation interface are shown in Figure 1. Following completion of the brain CTA, the neurologist used videoconferencing to directly interact with the patient and their family. This process involved the following six steps: (1) confirming the diagnosis of AIS, (2) assessing the patient and calculating the NIHSS score, (3) verifying the indications and contraindications for intravenous thrombolysis, (4) supervising tPA administration if indicated, (5) communicating directly with the patient’s family to explain the indications and risks of EVT, and (6) precoordinating with the neurointerventionalist at the CSC to prepare the operating room after obtaining consent.

In contrast, the standard referral process involved direct telephone communication between emergency physicians at the PSC and CSC. In this process, the PSC emergency physician independently decided on the administration of tPA, managed the thrombolysis procedure, and determined whether the patient was a potential EVT candidate.

Patients were allocated according to institutional workflow, with the standard referral process used during regular working hours and teleconsultation used during off-hours, including weekday nights and weekends.

### Statistical Analyses

Descriptive statistics were reported as means and SDs or medians and IQRs. Group differences were analyzed using the independent 2-tailed *t* test for normally distributed continuous variables, the Mann-Whitney *U* test for nonnormally distributed continuous variables, and the chi-square test for categorical variables. A *P* value of less than .05 was considered statistically significant.

We used independent *t* tests for the primary efficacy outcome, DIDO time and its components. The secondary outcomes, except for the functional outcomes, were analyzed using independent *t* tests or Mann-Whitney *U* tests for continuous variables and chi-square tests for categorical variables. Safety outcomes were compared using the chi-square test. To evaluate functional outcomes, we compared the shift in mRS from presentation to the PSC to the 90th day between groups using proportional odds logistic regression to calculate the odds ratio (OR) for mRS improvement, indicating the likelihood of shifting toward better functional outcomes. The distribution of mRS scores at presentation to the PSC and on the 90th day for each group was visualized using stacked bar charts.

## Results

### Participants and Baseline Characteristics

**Figure 2.**
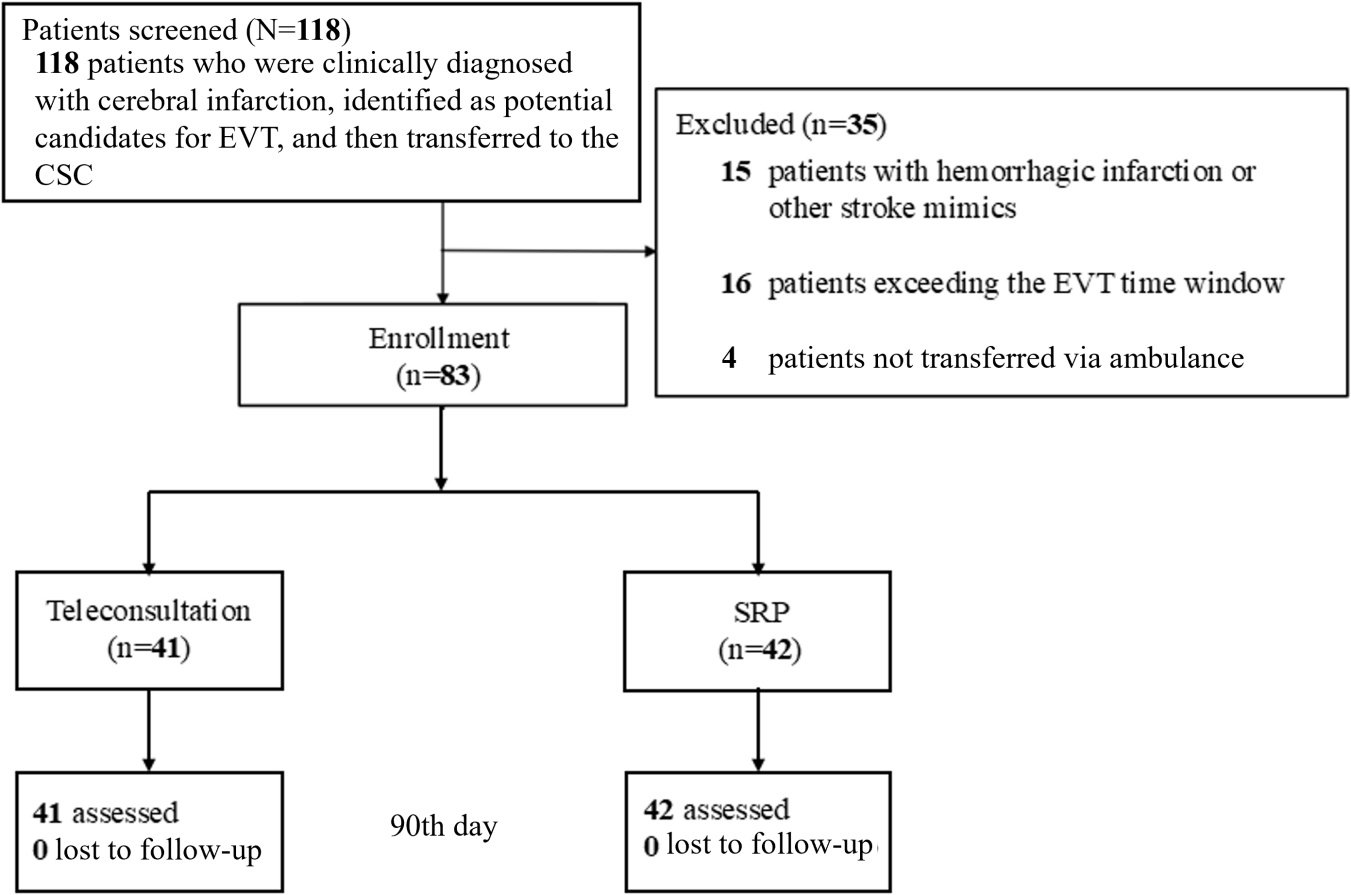
Study flow diagram. CSC: comprehensive stroke center; EVT: endovascular thrombectomy; SRP: standard referral process.

A total of 83 patients were enrolled, with 41 (49.4%) in the teleconsultation group and 42 (50.6%) in the standard referral process group (Figure 2). The mean age was 73.3 years, and the baseline characteristics were comparable between the 2 groups. There were no significant differences in gender, comorbidities, smoking, mRS at presentation to the PSC, or NIHSS scores (Table 1).

**Table 1.** Baseline characteristics.

	Teleconsultation (n=41)	SRP^a^ (n=42)	*P* value
Age (y), mean (SD)	75.2 (12.4)	71.4 (13.5)	.19^b^
Male sex, n (%)	18 (43.9)	23 (54.8)	.32^c^
Hypertension, n (%)	33 (80.5)	31 (73.8)	.47^c^
LDL^d^ (mg/dL), mean (SD)	93.5 (31.9)	95.3 (42.7)	.83^b^
Hyperlipidemia, n (%)	21 (51.2)	27 (64.3)	.59^c^
DM^e^, n (%)	17 (41.5)	17 (40.5)	.93^c^
HbA_1c_^f^ (mg/dL), mean (SD)	6.6 (1.5)	6.5 (1.3)	.70^b^
Smoking, n (%)	13 (31.7)	15 (35.7)	.70^c^
PAOD^g^, n (%)	3 (7.3)	3 (7.1)	.98^c^
NIHSS^h^ score (0-42), median (IQR)	15 (10-21)	15 (7-26)	.65^i^
mRS^j^ score (0-6) at presentation to the PSC^k^, median (IQR)	5 (4-5)	5 (3-5)	.37^i^
AF^l^, n (%)	13 (31.7)	14 (33.3)	.88^c^

a SRP: standard referral process.

b *P* value analyzed using the independent *t* test.

c *P* value analyzed using the Mann-Whitney *U* test.

d LDL: low-density lipoprotein.

e DM: diabetes mellitus.

f HbA_1c_: hemoglobin A_1c_.

g PAOD: peripheral arterial occlusive disease.

h NIHSS: National Institutes of Health Stroke Scale.

i *P* value analyzed using the chi-square test.

j mRS: modified Rankin Scale.

k PSC: primary stroke center.

l AF: atrial fibrillation.

### Primary Outcome

The DIDO time was significantly shorter in the teleconsultation group than in the standard referral process group (mean 95.2, SD 22.9 vs 132.3, SD 41.5 minutes; *P*<.001). Among the DIDO components, the CTA-to-notification time was significantly reduced in the teleconsultation group (mean 44.6, SD 17.4 vs 79.5, SD 37.6 minutes; *P*<.001). Other components, including door-to-CTA time and notification-to-departure time, showed no significant differences except for a borderline significance in the notification-to-departure time (mean 29.2, SD 3.3 vs 30.9, SD 3.9 minutes; *P*=.05; Table 2).

**Table 2.** Primary outcome: the door-in-door-out (DIDO) time and the DIDO components between groups.

	Teleconsultation (n=41), mean (SD)	SRP^a^ (n=42), mean (SD)	*P* value
DIDO time (min)	95.2 (22.9)	132.3 (41.5)	<.001^b^
Door-to-CTA^c^ time (min)	21.2 (5.4)	21.9 (4.2)	.54^b^
CTA-to-notification time (min)	44.6 (17.4)	79.5 (37.6)	<.001^b^
Notification-to-departure time (min)	29.2 (3.3)	30.9 (3.9)	.05^b^

a SRP: standard referral process.

b *P* value analyzed using the independent *t* test.

c CTA: computed tomography angiography.

### Secondary Outcomes

For stroke-related decision-making, the rates of tPA at the PSC (26/41, 63.4% vs 17/42, 40.5%; *P*=.04) and EVT at the CSC (14/41, 34.2% vs 6/42, 14.3%; *P*=.03) were higher in the teleconsultation group than in the standard referral process group. Among EVT-related factors in the CSC, the door-to-puncture time was shorter in the teleconsultation group (mean 83.0, SD 35.5 vs 118.5, SD 25.9 minutes; *P*=.04). The rate of tPA administration prior to EVT revealed no significant differences (9/14, 64.3% vs 4/6, 66.7%; *P*=.92). The reperfusion rate was higher in the teleconsultation group (13/14, 92.9% vs 3/6, 50%; *P*=.03; Table 3).

**Table 3.** Secondary outcomes and safety outcomes.

	Teleconsultation (n=41)	SRP^a^ (n=42)	*P* value
Stroke-related decision-making, n/N (%)			
Intravenous thrombolysis at the PSC^b^	26/41 (63.4)	17/42 (40.5)	.04^c^
EVT^d^ at the CSC^e^	14/41 (34.1)	6/42 (14.3)	.03^c^
EVT-related factors			
Door-to-puncture time (min), mean (SD)	83.0 (35.5)	118.5 (25.9)	.04^f^
Intravenous thrombolysis before EVT, n/N (%)	9/14 (64.3)	4/6 (66.7)	.92^c^
Reperfusion rate (mTICI^g^ score of >2b), n/N (%)	13/14 (92.9)	3/6 (50)	.03^c^
Safety outcomes, n/N (%)			
All-cause mortality	5/41 (12.2)	7/42 (16.7)	.56^c^
sICH^h^ after intravenous thrombolysis and/or EVT	3/31 (9.7)	2/19 (10.5)	.92^c^

a SRP: standard referral process.

b PSC: primary stroke center.

c *P* value analyzed using the Mann-Whitney *U* test.

d EVT: endovascular thrombectomy.

e CSC: comprehensive stroke center.

f *P* value analyzed using the independent *t* test.

g mTICI: modified treatment in cerebral infarction.

h sICH: symptomatic intracranial hemorrhage.

**Figure 3.**
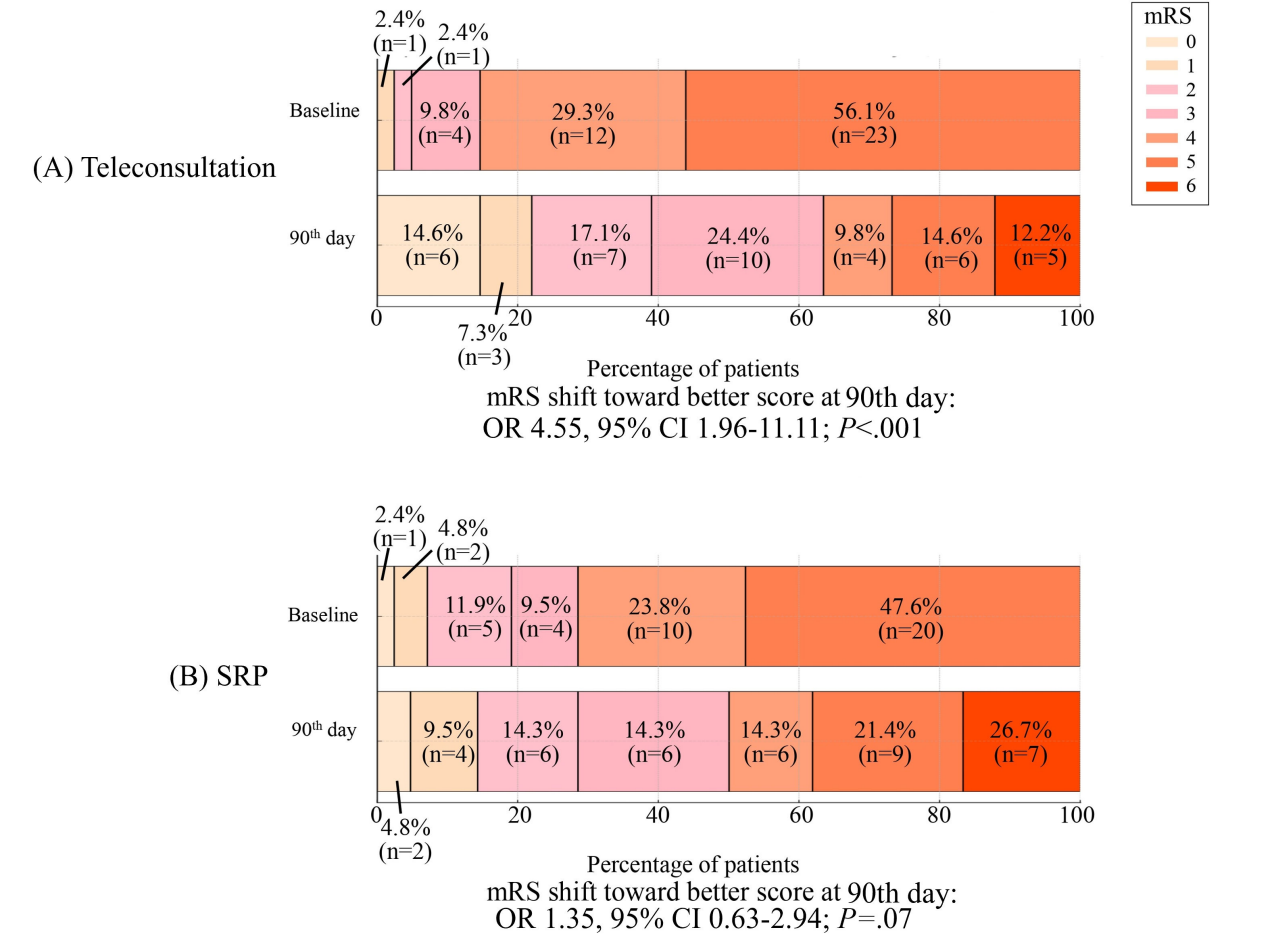
The modified Rankin Scale (mRS) score at presentation to the primary stroke center and the functional outcomes at the 90th day. OR: odds ratio; SRP: standard referral process.

Among functional outcomes, at the 90th day, the teleconsultation group demonstrated a greater shift toward better mRS scores (27/41, 65.9% of the participants; OR 4.55, 95% CI 1.96-11.11; *P*<.001) compared to the standard referral process group (13/42, 31.0% of the participants; OR 1.35, 95% CI 0.63-2.94; *P*=.07; Figure 3).

### Safety Outcomes

There was no significant difference in all-cause mortality within 90 days between groups (5/41, 12.2% vs 7/42, 16.7%; *P*=.56) or in the incidence of sICH after tPA and/or EVT (3/31, 9.7% vs 2/19, 10.5%; *P*=.92; Table 3).

## Discussion

### Principal Findings

Our study suggests that videoconferencing teleconsultation is associated with improved interhospital transfer efficiency and functional outcomes for patients with AIS within a hub-and-spoke model. The DIDO time, a critical metric for transfer efficiency, was significantly reduced in the teleconsultation group, driven primarily by a shorter CTA-to-notification time. During the study period, EVT eligibility was determined based on national health insurance reimbursement policies in Taiwan, which adopted extended time windows for anterior circulation stroke only after November 2023. To maintain consistency in inclusion criteria, patients beyond the previous time window were excluded.

Prior research, including a retrospective cohort study in a Dutch ambulance region, identified CTA-to-notification time as the largest contributor to total DIDO time, often exceeding 40% [10]. This delay is primarily attributed to the sequential nature of imaging interpretation, decision-making, and notifying the receiving center. Traditionally, these steps occur in isolation, resulting in cumulative delays before a final transfer decision is made. In our study, the significant reduction in CTA-to-notification time observed in the teleconsultation group may be related to the integration of real-time teleconsultation and 5G-enabled patient assessment, both of which likely contributed to a more streamlined workflow.

First, real-time teleconsultation allowed CSC neurologists to engage in the diagnostic process earlier, either while CTA was being performed or during the interval awaiting the formal CTA report. This parallel approach eliminated the traditional stepwise delays, allowing neurologists to simultaneously interpret imaging results, assess stroke severity, and discuss treatment plans with PSC emergency physicians. The ability to directly support PSC physicians in evaluating tPA eligibility and communicating with patients and their families may have facilitated faster decision-making and contributed to the higher tPA administration rate. This aligns with prior studies demonstrating that teleconsultation improves thrombolysis rates, particularly in facilities lacking on-site neurologists [13,14]. Second, the 5G-enabled patient assessment likely played a role in improving workflow efficiency by integrating real-time video and clinical data sharing. Unlike conventional workflows in which imaging review and patient assessment occur in separate sequential steps, this system enabled CSC neurologists to conduct simultaneous neurological evaluations and imaging interpretation, ensuring that NIHSS scoring and stroke severity assessments were completed without unnecessary delays [15]. By minimizing gaps between imaging interpretation and transfer notification, decision-making efficiency was likely enhanced [16]. Third, neurologists provided supervision of thrombolysis administration, ensuring protocol adherence and reducing procedural uncertainties, which may have contributed to optimizing treatment delivery and alleviating the workload of PSC physicians [17]. The combined effect of these improvements underscores the potential of videoconferencing teleconsultation in enhancing acute stroke management in resource-limited settings where on-site stroke expertise is unavailable.

Beyond facilitating initial treatment decisions at the PSC, teleconsultation was also associated with higher EVT rates and improved procedural efficiency at the CSC. Traditional telephone-based handovers are often fragmented, leading to delays in aligning treatment plans and preparing intervention teams [18]. In contrast, the video-based teleconsultation model enabled synchronized discussions between PSC and CSC teams, ensuring immediate procedural planning and early mobilization of neurointerventionalists [19]. This streamlined coordination likely contributed to the higher EVT rates, consistent with prior studies showing that pretransfer consultation increases EVT use. Furthermore, the reduction in door-to-puncture time suggests that early procedural activation played a crucial role in optimizing workflow efficiency. Teleconsultation enabled neurologists to assess imaging remotely before patient arrival, confirm EVT candidacy in advance, and initiate angiography suite preparations, leading to a more time-efficient transition. These findings align with evidence suggesting that early procedural planning and pretransfer activation of interventional teams are key determinants in reducing door-to-puncture time [19-21]. The improvement in reperfusion rates likely reflects the combined impact of increased EVT use and shorter procedural delays, which is highly time dependent [1,22].

Although teleconsultation has been well established to improve stroke-related decision-making, its effect on long-term functional outcomes remains controversial [8,23]. Prior studies have shown that hospitals with telestroke capacity achieve higher rates of reperfusion treatment and lower 30-day mortality than hospitals without telestroke capacity in rural settings [23]. As our PSC was located in a rural region, our findings reinforced the role of telestroke networks in expanding access to acute stroke therapies in rural stroke care. However, prior studies have not consistently demonstrated a direct association between telestroke network implementation and long-term functional improvement [5,8,24-27]. Notably, a previous study reported that telestroke network implementation did not significantly alter hospital transfer decisions, suggesting its primary role in improving treatment efficiency rather than hospital selection [28]. However, our current PSC-to-CSC model ensured that all eligible patients were transferred to the same CSC, eliminating confounding effects from variable hospital destinations. This allowed for a more precise evaluation of functional outcomes without interhospital transfer variability.

Our study provides additional evidence suggesting that videoconferencing teleconsultation may be associated with improved functional outcomes. Compared with prior studies that primarily focused on PSC-level decision-making, our study evaluated an integrated workflow spanning both a PSC and a CSC. By incorporating real-time video-based patient assessment, this model enables simultaneous clinical evaluation, imaging interpretation, and treatment planning. In addition to improving transfer efficiency, the observed association between workflow optimization and functional outcomes suggests a potential downstream clinical benefit of teleconsultation beyond conventional process measures. These findings are consistent with those of the TEMPiS (Telemedical Project for Integrative Stroke Care) trial [25], which demonstrated that telestroke networks were associated with a lower proportion of poor outcomes, reinforcing the benefits of telemedicine in acute stroke management. However, while prior research has primarily emphasized reducing severe disability and mortality, our study further explored functional outcome improvements. Taken together, our findings suggest that the potential benefit of teleconsultation may extend beyond isolated improvements in treatment rates to encompass system-wide optimization of stroke care pathways. Future research should investigate whether similar benefits can be observed in more diverse stroke networks.

Notably, while this study used a single hub–and–single spoke design, the streamlined and well-defined teleconsultation workflow described herein suggests that the model is highly feasible and potentially scalable. We have provided a detailed account of the process, encompassing early identification at the PSC, interhospital communication, and pretransfer decision-making. Given that implementation requires only a stable internet connection, standard videoconferencing tools, and a dedicated stroke team on call, this approach could be adapted in other underserved regions aiming to strengthen acute stroke care delivery through telemedicine.

Safety outcomes, including rates of sICH and all-cause mortality, were comparable between groups, supporting the safety of integrating teleconsultation into stroke care. In addition, the comparable rates of tPA administration prior to EVT suggest that the process does not compromise standard protocols but, instead, enhances patient selection and coordination for advanced therapies, which is in line with prior studies demonstrating that remote supervision of intravenous tPA via telemedicine is both feasible and safe [29].

Importantly, beyond clinical outcome measures, our study highlights the role of videoconferencing teleconsultation as a system-level digital health intervention that enables real-time, synchronized decision-making across geographically separated care settings. Unlike conventional telestroke models that primarily support decision-making at the PSC, our approach integrates pretransfer evaluation, interhospital communication, and procedural preparation at the CSC into a continuous workflow. This end-to-end coordination represents a key advancement in telemedicine-enabled stroke care, shifting from isolated decision support toward comprehensive workflow integration. Such a model may be particularly relevant for rural and resource-limited regions, where timely access to specialized stroke care remains a major challenge.

The strengths of this study include a detailed analysis of DIDO time, allowing for precise identification of workflow inefficiencies. The integration of real-time videoconferencing facilitated real-time patient assessment, ensuring direct NIHSS scoring and verification of tPA eligibility. Furthermore, the PSC in our study serves as the primary stroke referral center for multiple rural townships in central Taiwan, including Puli, Guoxing, Ren’ai, Yuchi, and Zhongliao, with a total service population of approximately 150,000. Our findings may offer applicable insights for enhancing rural stroke care.

However, several limitations should be considered. First, the relatively small sample size limits the statistical power of this study and may affect the precision of effect estimates, particularly for secondary and safety outcomes. As a result, this study may be underpowered to detect modest differences between groups, and the observed associations, especially regarding functional outcomes, should be interpreted with caution. Second, the single hub–and–single spoke design, chosen to minimize variability in transfer times by ensuring that all patients were transported via ambulance, may further limit the generalizability of our findings to more complex, multicenter stroke networks. Third, the retrospective design might introduce potential selection bias as treatment decisions were based on real-world clinical practice rather than on randomized allocation. Fourth, the inclusion criteria were aligned with the reimbursement policy of the Taiwan National Health Insurance system, which covers EVT within 6 hours for anterior circulation stroke and within 24 hours for posterior circulation stroke. This policy-driven framework may have resulted in the inclusion of patients who were transferred but ultimately did not undergo EVT. In addition, group allocation was determined by institutional workflow, which may introduce selection bias. Notably, workflow efficiency remained improved in the teleconsultation group despite being implemented during off-hours.

### Conclusions

Videoconferencing teleconsultation was associated with improved interhospital transfer efficiency, facilitated timely reperfusion therapy, and may be associated with better functional outcomes in patients with AIS within a hub-and-spoke model while maintaining patient safety. Future research should evaluate this model’s scalability in multicenter networks and its impact on broader stroke populations beyond standard EVT eligibility criteria.

## Abbreviations

AIS: acute ischemic stroke
CSC: comprehensive stroke center
CTA: computed tomography angiography
DIDO: door-in-door-out
EVT: endovascular thrombectomy
mRS: modified Rankin Scale
NIHSS: National Institutes of Health Stroke Scale
OR: odds ratio
PSC: primary stroke center
sICH: symptomatic intracranial hemorrhage
TEMPiS: Telemedical Project for Integrative Stroke Care
tPA: tissue-type plasminogen activator
